# Minocycline partially reverses established PTSD-related behavioral traits in rats exposed to repetitive low-level blast injury

**DOI:** 10.3389/fneur.2025.1666737

**Published:** 2025-12-02

**Authors:** Georgina Pérez Garcia, Gissel M. Perez, Rita De Gasperi, Miguel A. Gama Sosa, Rania Abutarboush, Usmah Kawoos, Carolyn Zhu, Carlos A. Toro, Patrick R. Hof, Stephen T. Ahlers, Gregory A. Elder

**Affiliations:** 1 Research and Development Service, James J. Peters Department of Veterans Affairs Medical Center, Bronx, NY, United States; 2 Department of Neurology, Icahn School of Medicine at Mount Sinai, New York, NY, United States; 3 Department of Psychiatry, Icahn School of Medicine at Mount Sinai, New York, NY, United States; 4 General Medical Research Service, James J. Peters Department of Veterans Affairs Medical Center, Bronx, NY, United States; 5 Department of Neurotrauma, Naval Medical Research Command, Silver Spring, MD, United States; 6 The Henry M. Jackson Foundation for the Advancement of Military Medicine Inc., Bethesda, MD, United States; 7 Mount Sinai Alzheimer's Disease Research Center and Ronald M. Loeb Center for Alzheimer's Disease, Icahn School of Medicine at Mount Sinai, New York, NY, United States; 8 Spinal Cord Damage Research Center, James J. Peters Department of Veterans Affairs Medical Center, Bronx, NY, United States; 9 Department of Medicine, Icahn School of Medicine at Mount Sinai, New York, NY, United States; 10 Nash Family Department of Neuroscience and Friedman Brain Institute, Icahn School of Medicine at Mount Sinai, New York, NY, United States; 11 Department of Geriatrics and Palliative Care, Icahn School of Medicine at Mount Sinai, New York, NY, United States; 12 Neurology Service, James J. Peters Department of Veterans Affairs Medical Center, Bronx, NY, United States

**Keywords:** blast, minocycline, post-traumatic stress disorder, rat, traumatic brain injury

## Abstract

**Introduction:**

Many military Veterans who experienced blast-related traumatic brain injuries (TBI) in the conflicts in Iraq and Afghanistan currently suffer from chronic cognitive and mental health problems including post-traumatic stress disorder (PTSD). Rats exposed to repetitive low level blast injury exhibit chronic PTSD-related behavioral traits. Inflammation has long been suspected of playing a role in blast-induced brain injury and rats exposed to repetitive low-level blast develop chronic inflammatory changes. Minocycline is a tetracycline antibiotic that besides having antibacterial properties has anti-inflammatory activity. The aim of this study was to determine whether minocycline could reverse PTSD related behavioral traits in rats exposed to repetitive low level blast exposure.

**Methods:**

Rats were exposed to three 74.5 kPa blast exposures administered one per day for three consecutive days. We tested two cohorts of blast-exposed rats at 8–8.5 months after blast exposure. Rats were tested in a novel object recognition (NOR) task, elevated zero maze (EZM) and cued fear learning paradigm. In one experiment rats were treated with five doses of minocycline over a 9-day period. In the second experiment blast-exposed rats were treated with a 4-week course of minocycline with the drug administered 11 times. After the second experiment blast-induced effects on expression of the serotonin receptor 2A (5-HT2AR) and the post-synaptic density protein-95 (PSD-95) were examined by Western blotting. Microglial morphology was examined by Iba1 immunostaining.

**Results:**

In both experiments, cognitive changes in NOR and anxiety in an EZM were reversed by minocycline. However, in neither experiment was exaggerated fear learning rescued. Minocycline did not reverse blast induced effects on expression of 5-HT2AR or PSD-95 although it did appear to modulate blast-induced effects on microglial morphology.

**Conclusions:**

These studies have implications for understanding the nature of blast-induced behavioral traits, some of which may be the direct result of inflammatory effects, while others may be independent of inflammation or if the result of inflammation, not reversible once downstream structural or neurochemical changes are established.

## Introduction

Military-related traumatic brain injuries (TBIs) occur for many reasons, but certain types are relatively unique to military environments. The most prominent of these is blast exposure. In Iraq and Afghanistan, exposure to improvised explosive devices (IEDs) caused most TBIs in Service Members (1). Concern also exists over the possible effects of subclinical blast exposure (2). Military occupational blast exposure as it is now being referred to is common for many Service Members during training and military operations. Whether repetitive, low-level blast exposure causes health problems in later life is unclear, but cumulative low-level blast exposure over a Veteran's career has been associated with chronically worse brain health, more post-traumatic stress disorder (PTSD), and a greater risk for developing new symptoms after a later blast injury (3).

PTSD is a mental health disorder that develops after experiencing or witnessing a psychologically traumatizing event (4). Clinical features include re-experiencing phenomena (e.g., flashbacks), as well as hyperarousal symptoms including heightened acoustic startle and avoidance of places or events that serve as reminders of the psychological trauma. Changes in mood and cognition are associated features.

Rats exposed to repetitive low-level blast develop PTSD-related traits including anxiety, increased acoustic startle and changes in cognition that appear in a delayed manner and remain present for more than 1 year after exposure (5–10). Blast-exposed rats also exhibit exaggerated fear learning which has been extensively studied in rodents for its relevance to the neurobiology of PTSD (11, 12). We have referred to the condition in these animals as blast-induced PTSD (7) in the belief that it models a state which may be different pathophysiologically from PTSD following a psychological stressor. These animals thus represent a model to study the chronic neurobehavioral syndromes that often affect Veterans after blast exposure (13–15). The progressivity seen in this model is reminiscent of that seen in longitudinal studies of Service Members who suffered blast-related TBIs in Afghanistan which found that between 1 and 5 years of follow up, overall global functioning declined in >70% a decline nearly completely driven by worsening PTSD and depression (14).

Inflammation has long been suspected of playing a role in blast-induced brain injury (16, 17). In animal models, blast exposure causes increases in many inflammatory markers in blood and plasma (16, 17). Elevated pro-inflammatory cytokines and chemokines as well as microglial activation have been observed in multiple brain regions (reviewed in Ref. (16, 17)). Recently, a study in special forces personnel identified brain inflammation using a positron emission tomography ligand which recognizes activated microglia (18). The increases correlated with lifetime blast exposure (18).

Rats exposed to repetitive blast exposure in our model exhibit an early and selective vascular pathology with prominent gliovascular injury (17, 19, 20). Perivascular inflammation is a chronic feature of the disease (21) and associated with chronic vascular remodeling as well as increased levels of matrix metalloproteinases (MMP-2 and MMP-9), collagen IV loss, and microglial activation around affected vasculature. A transcriptomic study in our rat model (22) identified tumor necrosis factor α (TNFα) as a potential upstream regulator of a cluster of differentially expressed genes (DEGs) whose expression was affected by blast exposure across the time frame over which the neurocognitive phenotype appears.

Minocycline has broad anti-inflammatory and neuroprotective actions (23) with multiple studies documenting its ability to inhibit TNFα production (24–30). Minocycline has been widely studied for treatment of TBI in animals with Bergold et al. in their 2023 review identifying over 40 studies in mice and rats (31). These studies which have been conducted almost exclusively in the acute setting and in non-blast models suggest that minocycline has significant neuroprotective effects acutely (31). In one study using a blast model (32) rats were treated for four consecutive days with 50 mg/kg minocycline after a single blast injury. At 8 and 45 days after blast exposure, behavior in blast-exposed animals treated with minocycline was nearly identical to controls while several serum and tissue level markers of inflammatory, as well as vascular, neuronal, and glial markers of injury were normalized (32). Another study found that minocycline delivered via nanoparticles could ameliorate blast induced hearing loss in an animal model (33). In the present study, we tested whether minocycline could reverse established PTSD related behavioral traits in a well-characterized rat model of repetitive low level blast exposure.

## Methods

### Animals

A total of 73 adult male Long Evans hooded rats (250–350 g; 10 weeks of age; Charles River Laboratories International, Wilmington, MA, USA) were used. All studies involving animals were approved by the Institutional Animal Care and Use Committees of the Walter Reed Army Institute of Research (WRAIR)/Naval Medical Research Command (NMRC) and the James J. Peters VA Medical Center. Studies were conducted in compliance with the Public Health Service policy on the humane care and use of laboratory animals, the NIH Guide for the Care and Use of Laboratory Animals, and all applicable Federal regulations governing the protection of animals in research.

### Blast overpressure exposure

Rats were exposed to overpressure injury using a shock tube at either the WRAIR/NMRC (cohort 1) or the James J. Peters VA Medical Center (cohort 2). Exposures using the WRAIR/NMRC shock tube have been described in detail in multiple prior studies (5–9, 19–22, 34–38). The shock tube at the James J. Peters VA Medical Center was designed and constructed by Baker Engineering and Risk Consultants (San Antonio, TX, USA). The instrument is approximately 6.4 m in length and comparable in design to other shock tubes in use that have been built by Baker Risk (39). The tube consists of a variable volume driver that permits control of the duration of the primary positive pressure wave independent of the peak overpressure. Pressurized air was used for all experiments. A dual diaphragm spooler holding two polyethylene terephthalate Mylar TM sheets (Du Pont, Wilmington, DE, USA) was used to control pressure differences between the driver and expansion section of the tube. To induce “detonation,” the pressure between the two diaphragms of the spooler was released via remotely controlled electronic valves causing both diaphragms to rupture simultaneously. The peak pressure at the end of the expansion chamber was determined with piezoresistive gauges specifically designed for pressure-time (impulse) measurements (Model 102M152, PCB, Piezotronics, Depew, NY, USA). All sensor data was collected and processed using Lab View2011 software (Austin, TX, USA). The end of the shock tube is fitted with an attenuator chamber that reduces ambient blast noise and suppresses reflected shock waves.

Individual rats were anesthetized using an isoflurane gas anesthesia system consisting of a vaporizer, gas lines and valves and an activated charcoal-scavenging system adapted for use with rodents. Rats were placed into a polycarbonate induction chamber, which was closed and immediately flushed with a 5% isoflurane mixture in air for 2 min. Rats were placed into a cone-shaped plastic restraint device and mounted on a square grid. Head and body movement was restricted by harnesses that affixed the animal to the grid and restricted movement during the blast overpressure exposure without restricting breathing. Rats were randomly assigned to sham or blast conditions and were placed in the shock tube lying prone with the plane representing a line from the tail to the nose of the body in line with the longitudinal axis of the shock tube and the head placed upstream facing the shock wave. The total length of time under anesthesia including placement in the shock tube and execution of the blast procedure was typically less than 3 min. Blast-exposed animals received 74.5 kPa peak overpressure exposures (equivalent to 10.8 psi, duration 4.8 ms, impulse 175.8 kPa^*^ms). These exposures closely mimic those used in prior studies which used the WRAIR/NMRC shock tube (40). Exposures were administered one exposure per day for three consecutive days.

### Animal housing and handling

Animals were housed at a constant 21–22 °C temperature with rooms on a 12:12 hour light cycle with lights on at 7 a.m. All subjects were individually housed in standard clear plastic cages equipped with Bed-O'Cobs laboratory animal bedding (The Andersons, Maumee, OH, USA) and EnviroDri nesting paper (Sheppard Specialty Papers, Milford, NJ, USA). Access to food and water was *ad libitum*. Subjects were housed on racks in random order to prevent rack position effects. Prior to behavioral testing, animals were habituated to handling to reduce stress during subsequent experimental procedures. Subjects were gently removed from their home cages and allowed to freely explore an open area measuring 100 cm × 60 cm for 5 min, three times per week. Following this, researchers gradually introduced handling by gently restraining the animals to further acclimate them to human manipulation. Cages were coded to allow maintenance of blinding to groups during behavioral testing. Experimenters were blinded to experimental groups to prevent bias during handling and data collection.

### Drug administration

**Figure 1:**
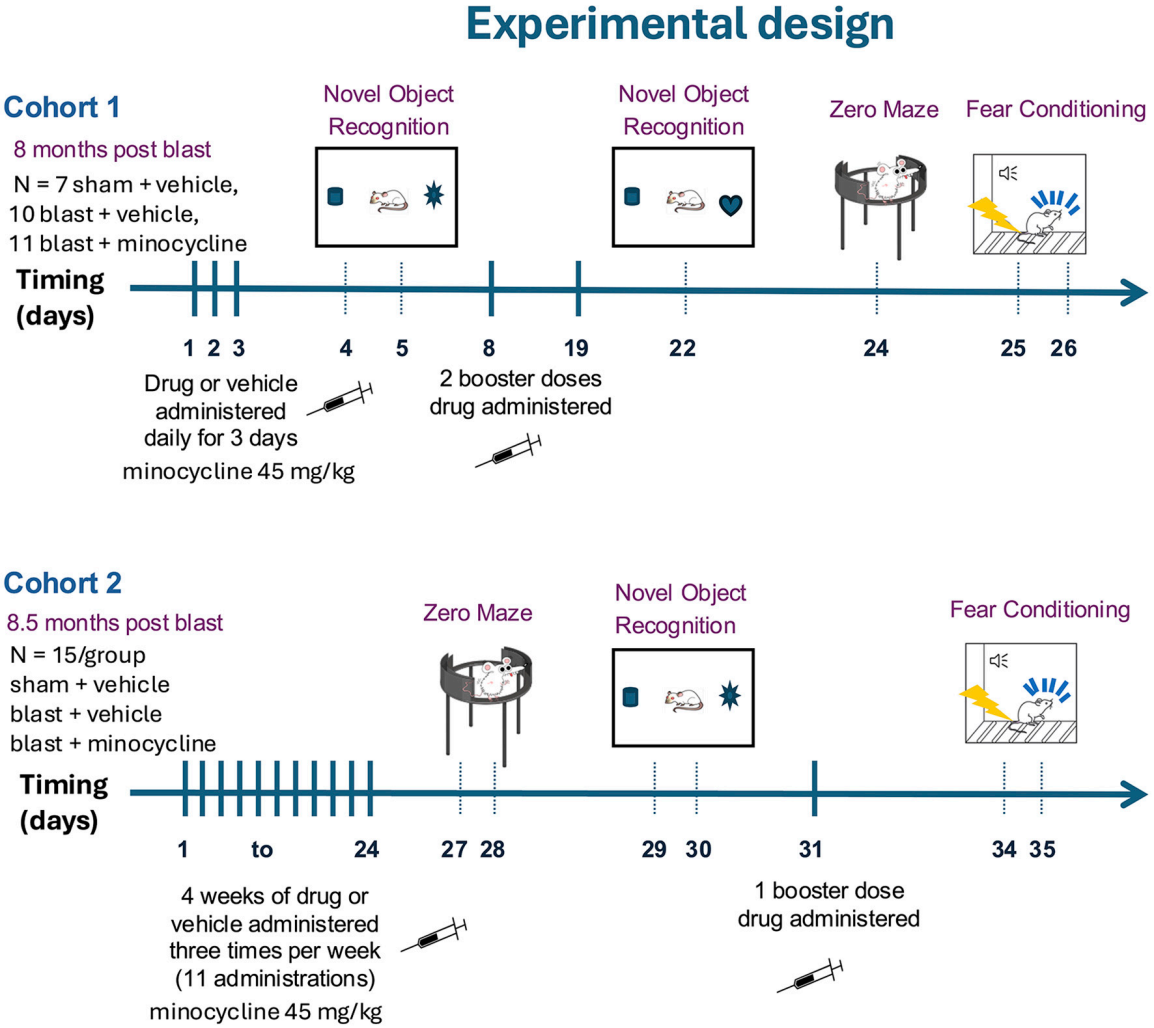
Experimental design. Minocycline (45 mg/kg) was administered to two cohorts of blast-exposed rats. Cohort 1 (*n* = 7 sham + vehicle, 10 blast + vehicle, 11 blast + minocycline) was treated at 8 months after blast exposure. Administrations of minocycline were given daily for three consecutive days. On day 4 and 5 rats were tested in an NOR task. Rats were given additional booster doses of minocycline on days 8 and 19. On day 22 they were retrained and tested in NOR. On day 24 they were tested in an elevated zero maze (EZM) and on days 25 and 26 trained and tested in a cued fear conditioning protocol. Cohort 2 (*n* = 15 per group, sham + vehicle, blast + vehicle and blast + minocycline) was treated at 8.5 months after blast exposure. Rats received three treatments per week with the first administration on a Wednesday and then continued Monday, Wednesday, Friday over a period of 4 weeks (11 administrations total over days 1–24). On days 27 and 28 they were tested in an EZM and then on days 29 and 30 in an NOR task. On day 31 they were given one additional booster dose of drug or vehicle. On days 34 and 35, they were trained and tested in a cued fear conditioning protocol.

Minocycline (Sigma, St. Louis MO, USA) was dissolved in saline. Blast exposed subjects were treated with 45 mg/kg per administration given intraperitoneally. Animals were divided into three experimental groups: (1) sham-exposed rats treated with vehicle (sham + vehicle) (2) blast-exposed rats treated with vehicle (blast + vehicle), and (3) blast-exposed rats treated with minocycline (blast + Mino). Two cohorts were studied. The timing of drug administration in relationship to blast exposure and behavioral testing is shown in Figure 1. Dose was chosen based on 45 mg/kg being a dose that is tolerated and has successfully improved outcomes in multiple rat models of TBI (31).

### Behavioral testing

The exact timing of testing of each cohort in relationship to blast exposure and drug administration is described in Figure 1.

#### Elevated zero maze (EZM)

The apparatus consisted of a circular black Plexiglas runway 121.92 cm in diameter and raised 76 cm off the floor (San Diego Instruments, San Diego, CA, USA). The textured runway itself was 5.08 cm across and divided equally into alternating quadrants of open runway enclosed only by a 1.27 cm lip and closed runway with smooth 15.24 cm walls. All subjects received a 5-min trial beginning in a closed arc of the runway. During each trial, subjects were allowed to move freely around the runway, with all movement tracked automatically by a video camera placed on the ceiling directly above the maze. Data were analyzed by ANYMAZE (San Diego Instruments) yielding measures of total movement time and distance for the entire maze, as well as time spent, and distance traveled in each of the individual quadrants. From the quadrant data, measures of total open and closed arc times, latency to enter an open arc, total open arm entries and latency to completely cross an open arc between two closed arcs were calculated. Subject position was determined by centroid location.

#### Novel object recognition (NOR)

Rats were habituated to the arena (90 cm length × 60 cm width × 40 cm height) for 20 min, 24 h before training. On the training day, two identical objects were placed on opposite ends of the empty arena, and the rat was allowed to explore the objects freely for 7 min. After a 1-h delay, during which the rat was held in its home cage, one of the two familiar objects was replaced with a novel one, and the rat was allowed to freely explore the familiar and novel object for 5 min to assess short-term memory (STM). After a 24-h delay, during which the rat was held in its home cage, the novel object from the STM testing was replaced with a second novel object different from the one used during STM testing but placed in the same location as the novel object in the STM testing. The rat was allowed to freely explore the familiar and novel object for 5 min to assess long-term memory (LTM). Raw exploration times for each object were expressed in seconds. Object exploration was defined as sniffing or touching the object with the vibrissae or when the animal's head was oriented toward the object with the nose placed at less than 2 cm from the object. All sessions were recorded by video camera (Sentech, Carrollton TX, USA) and analyzed with ANYMAZE software (San Diego Instruments). In addition, offline analysis by an investigator blind to the blast-exposed status of the animals was performed. Objects to be discriminated were of different size, shape and color and were made of plastic or metal material. The objects consisted of a 330 ml soda can, a metal box, a cup and a plastic tube. All objects were cleaned with 70% ethanol between trials.

### Cued fear conditioning

Sound-attenuated isolation cubicles (Ugo Basile, Gemonio, (VA), Italy) were utilized. Each cubicle was equipped with a grid floor for delivery of the unconditioned stimulus (US) and overhead cameras. All aspects of the test were controlled and monitored by the Freeze Frame conditioning and video tracking system (Ethovision, Leesburg, VA, USA). During training the chambers were scented with almond extract, lined with white paper towels, had background noise generated by a small fan and were cleaned before and between trials with 70% ethanol. Each subject was placed inside the conditioning chamber for 2 min before the onset of a conditioned stimulus (CS; an 80 dB, 2 kHz tone), which lasted for 20 s with a co-terminating 2-s footshock [0.7 mA; unconditioned stimulus (US)]. A total of three tone/shock pairings were administered with the first/second and second/third separated by 1 min. Each rat remained in the chamber for an additional 40 s following the third CS-US pairing before being returned to its home cage. Freezing was defined as a lack of movement (except for respiration) in each 10-s interval. Minutes 0–2 during the training session were used to measure baseline freezing. Animals were returned to their home cage for another 24 h at which time cued fear response was tested. To create a new context with different properties, the chambers were free of background noise (fan turned off), lined with blue paper towels, scented with lemon extract and cleaned before and during all trials with isopropanol. Each subject was placed in this novel context for 2 min and baseline freezing was measured, followed by exposure to the CS (20-s tone) at 120, 260 and 420 s.

### Tissue collection

For biochemical studies rats were euthanized by CO_2_ inhalation, the brain was removed and regionally dissected as previously described (5). For immunohistochemistry, rats were perfused with 4% paraformaldehyde (PFA) in phosphate buffer saline (PBS) and the brain dissected and post-fixed overnight in PFA and stored in PBS.

### Western blot analysis

Tissues were homogenized in 0.1 M Tris HCl pH 7.6 containing 150 mM NaCl, 1% Triton, 0.1% and a protease and phosphatase inhibitors (Halt, Protease and phosphatase inhibitor cocktail, ThermoFisher, Waltham MA, USA) using Zirconia beads and the Fast prep tissue homogenizer (MP Biomedicals, Irvine, CA, USA). The lysates were centrifuged at 14,000 rpm for 20 min and the supernatant saved and frozen at −80 °C. Protein concentration was measured with the BCA assay (ThermoFisher). Protein (50 μg) was separated on SDS-PAGE gels, blotted onto Immobilon P membranes (Millipore) and blocked in a 5% non-fat dry milk in 20 mM Tris HCl, pH 7.5, 0.25 M NaCl, 0.1% Tween 20 (TBST). The membrane was incubated overnight at 4 °C with the primary antibody (Table 1) diluted in blocking solution, washed in TBST and incubated at room temperature 1.5 h with horseradish conjugated anti rabbit IgG (NA934, 1:7,500, Cytiva, Marlborough, MA, USA) diluted in blocking solution. The membrane was developed with ECL Prime western blotting detection reagent (Cytiva) and imaged with Amesham ImageQuant 800 imaging system. Membranes were stained with Ponceau Red S Stain (Sigma) to image total protein loading. Bands were quantitated with ImageQuant TL (Cytiva). Levels were expressed as ratio to glyceraldehyde 3- phosphate dehydrogenase (GAPDH) or to total protein loading derived from integrated intensity of the Ponceau S-stained bands. Stripping of blots was performed with Reblot Plus buffer (ThermoFisher) as recommended by the manufacturer.

**Table 1:** Antibodies used for western blotting.

**Antibody target**	**Type**	**Source**	**Catalog number**	**Dilution**
PSD-95	Rabbit monoclonal	Cell Signaling Technology	3409S	1:1,200
GAPDH	Rabbit monoclonal	Cell Signaling Technology	5174S	1:1,000
5-HT2AR	Mouse monoclonal	Santa Cruz	sc-166775	1:600

### Immunohistochemistry

Coronal sections (50 μm thickness) were prepared with a VT1000S Vibratome (Leica Biosystems, Buffalo Grove, IL, USA). Floating sections were blocked with 10% normal goat serum in 50 mM Tris HCl, pH 7.6, 0.15 M NaCl, 0.3% Triton-X-100 for 2 h and incubated overnight with rabbit anti-ionized calcium-binding adaptor molecule 1 polyclonal antibody (Iba1, 019-19741, RRID:AB_839504, Fujifilm Wako Pure Chemical, Osaka, Japan) diluted 1:300 in blocking solution at room temperature. After washing with PBS (six times for 10 min each), sections were incubated with an Alexa Fluor 647-conjugated anti-rabbit secondary antibody (1:300, ThermoFisher) in blocking solution for 2 h. To visualize nuclei, sections were incubated in 0.1 mg/ml DAPI 4′,6-diamidine-2′-phenylindole dihydrochloride) in PBS. After washing with PBS (six times for 10 min each), the sections were mounted with Fluorogel mounting medium (Electron Microscopy Sciences, Hatfield, PA, USA). Immunostained sections were imaged with a laser scanning confocal microscope Zeiss LSM 980 with Airyscan 2 (Carl Zeiss Microscopy, White Plains, NY, USA).

### Statistical analysis

Data sets were examined for assumptions of parametric tests (normality, homogeneity of variance). Values are expressed as mean ± SEM. Comparisons were performed using univariate ANOVA, repeated-measures ANOVA, or unpaired *t*-tests. When three groups were being compared if the ANOVA was significant (*p* ≤ 0.05), Fisher's LSD was used to determine between group differences. When repeated-measures ANOVA was used the Greenhouse-Geisser correction was utilized. Statistical tests were performed using the program GraphPad Prism 10.6.1 (GraphPad Software, San Diego, CA, USA) or SPSS v30 (IBM, Chicago IL, USA).

## Results

### Short term treatment with minocycline partially reverses blast induced neurobehavioral deficits

**Figure 2:**
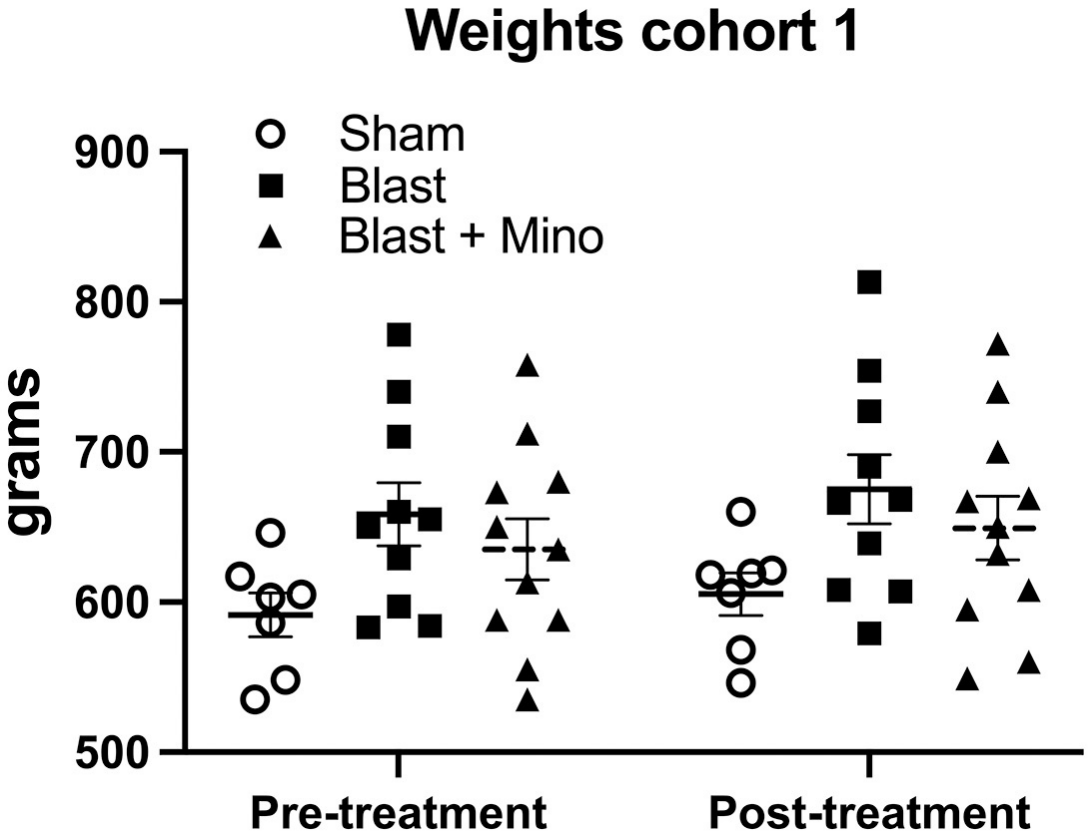
Weights for cohort 1. Shown are the weights for the rats used in cohort 1, obtained before treatment and at the end of behavioral testing. A repeated measures ANOVA showed that rats gained weight from before treatment to after behavioral testing (*F*_1, 25_ = 7.535, *p* = 0.011) but there was no interaction effect of group (*F*_2, 25_= 0.024, *p* = 0.976) and no between group differences (*F*_2, 25_ = 2.537, *p* = 0.099). Error bars indicate the standard error of the mean (SEM).

To determine whether minocycline might reverse blast-induced effects on behavior we treated blast-exposed rats at 8 months after blast exposure with 45 mg/kg of minocycline for 5 days over a 9-day period (Figure 1, cohort 1). A three-group design was used in which sham-exposed rats were treated with vehicle alone while groups of blast-exposed rats were treated with vehicle or minocycline. Active drug or vehicle were administered intraperitoneally. In this design comparison of sham- and blast-treated with vehicle served as a positive control for the presence of the blast-induced behavioral phenotype while comparison of blast-exposed treated with minocycline vs. blast-treated with vehicle allowed effect of minocycline to be determined. The weights for cohort 1 before treatment and at the end of behavioral testing are shown in Figure 2. There were no between group differences (*F*_2, 25_ = 2.537, *p* = 0.099) and there was no mortality during the study.

**Figure 3:**
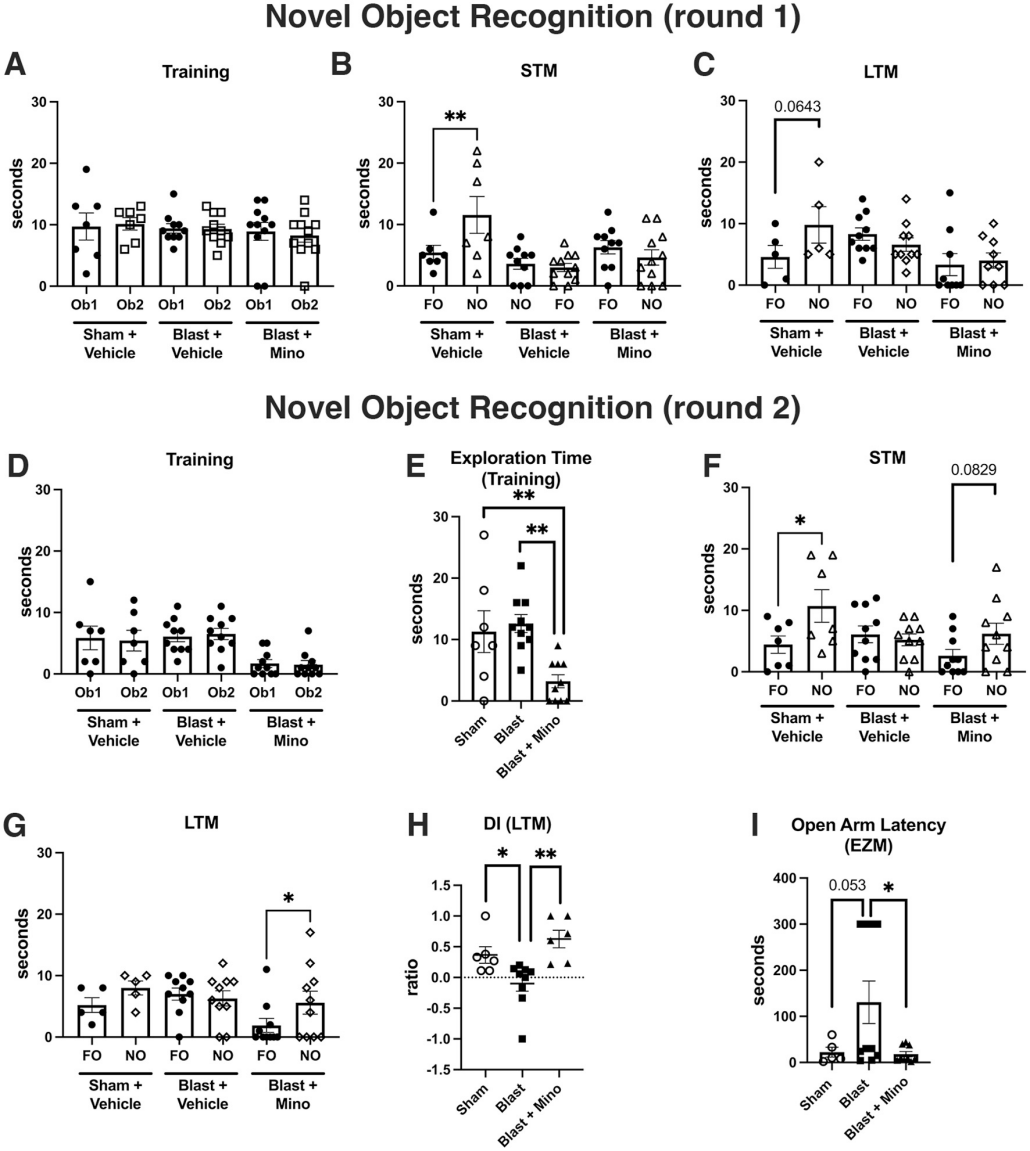
Testing of cohort 1 in novel object recognition (NOR) and elevated zero maze (EZM). We treated blast-exposed rats at 8 months after blast exposure with 45 mg/kg of minocycline on days 1, 2, and 3 of the protocol shown in Figure 1. NOR testing was performed on days 4 and 5. Shown is time spent exploring object 1 (Ob1) or object 2 (Ob2) during the training **(A)**, short term memory testing (STM) at 1 h after training **(B)** or long-term memory testing (LTM) at 24 h after training **(C)**. Time spent exploring the familiar (FO) and novel (NO) objects were compared using unpaired *t*-tests (**p < 0.01). On days 8 and 19 of the protocol rats were administered additional doses of minocycline. On days 22 and 23 rats were tested again in an NOR task (round 2). Shown is training **(D)**, total exploration time during the training session **(E)**, testing for STM **(F)** and LTM **(G)** memory and a discrimination index (DI) calculated for the LTM testing **(H)**. Asterisks in **(B)**, **(F)** and **(G)** indicate comparisons using unpaired *t*-tests (*p < 0.05, **p < 0.01). For total exploration time **(E)**, a one-way ANOVA was significant (*F*_2, 24_ = 7.7, *p* = 0.0025). Asterisks indicate significant differences between groups (**p < 0.01, Fisher's LSD). On day 24 EZM testing was performed. Panel **(I)** shows the latency to enter an open arm. A one-way ANOVA was significant (*F*_2, 21_ = 3.8, *p* = 0.0374, *p < 0.05, Fisher's LSD). Error bars indicate the SEM in all panels.

After an initial 3 days of treatment, we tested rats on an NOR task (Figures 3A–C). In the short-term memory (STM) and long-term memory (LTM) testing at 1 h or 24 h after training the sham exposed rats spent more time exploring the novel object (NO) than the familiar object (FO). By contrast the blast-exposed rats treated with vehicle or minocycline spent an equal amount of time exploring the FO and NO indicating that blast-induced deficits in NOR were not rescued by minocycline.

Reasoning that the treatment had not been long enough, we treated these same rats with two additional doses of minocycline on days 8 and 19 (Figure 1). On day 22 of the protocol, rats were trained and tested again in an NOR task, on day 24 an EZM, and on days 25 and 26 trained and tested for cued fear learning. As shown in Figure 3D, in NOR testing all groups spent equal amounts of time exploring the objects in the training phase although the blast-treated with minocycline spent less total time exploring the objects (Figure 3E). When recognition memory was tested 1 h later (Figure 3F), sham-treated with vehicle explored the NO more than the FO (*p* < 0.05) while blast-exposed treated with vehicle did not explore the NO more than the FO. Blast-exposed treated with minocycline explored the NO more than the FO although this did not reach statistical significance (*p* = 0.08). However, when recognition memory was tested 24 h later (Figure 3G), the blast-exposed treated with minocycline explored the NO more than the FO (*p* < 0.05) while the sham- and blast-treated with vehicle explored them equally. When a discrimination index (DI), which measures the relative preference for the NO vs. FO, was calculated (Figure 3H), the sham-treated with saline preferred the NO to the FO more than the blast-exposed treated with vehicle. Preference for the NO was restored to that of sham + vehicle in blast-treated with minocycline. During the second round of behavioral testing, compared to controls, minocycline also reduced the latency of blast-exposed rats to enter an open arm in the EZM (Figure 3I).

**Figure 4:**
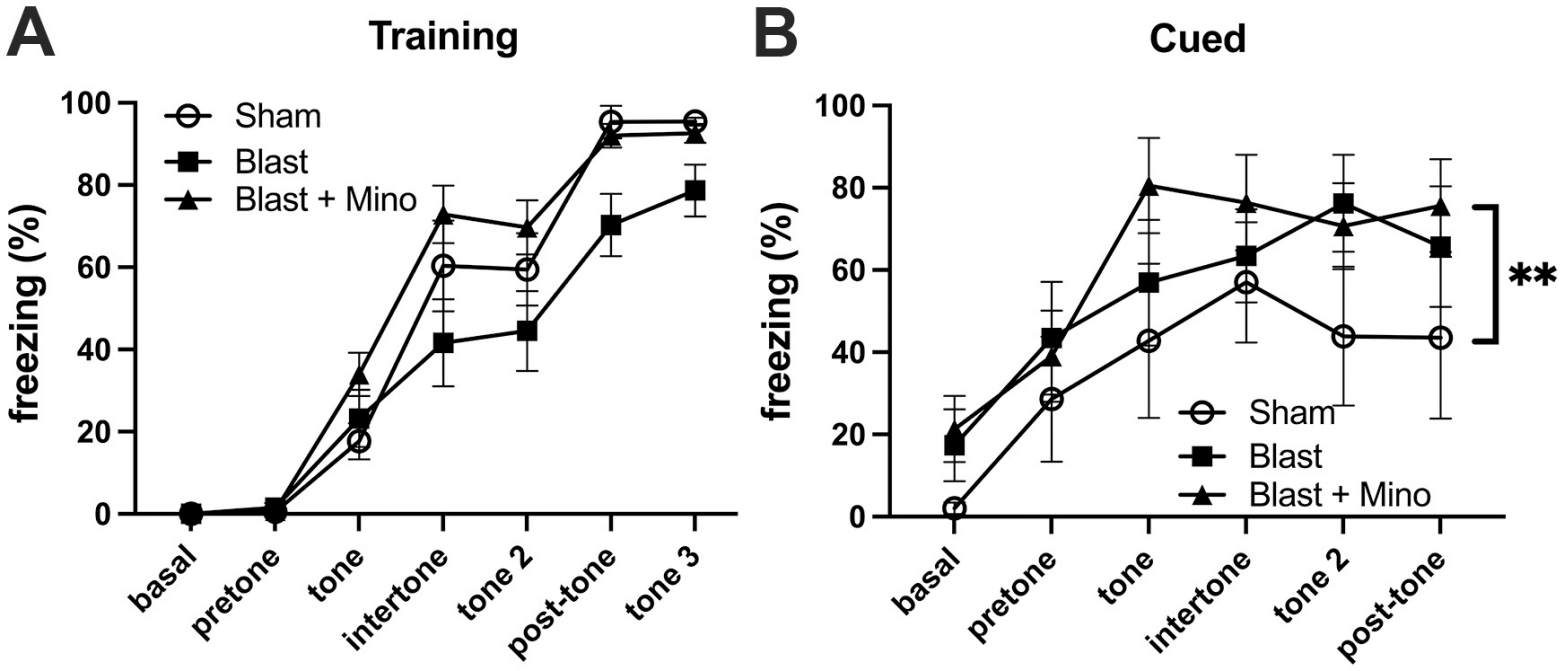
Testing of cohort 1 in fear learning. Panel **(A)** shows training. Basal and pretone represent the first and second minute respectively before the first presentation of the tone + shock. A repeated measures ANOVA showed that all groups responded with increasing freezing following pairing of the tone and shock (*F*_2.684, 53.681_ = 143.973, *p* < 0.001) with no interaction effect of group (*F*_5.368, 53.681_ = 2.176, *p* = 0.066) and no between group differences (*F*_2, 20_ = 2.084, *p* = 0.151). During the cued testing session **(B)**, all groups responded with freezing following presentation of the tone (repeated measures ANOVA: *F*_2.353, 35.295_ = 14.088, *p* < 0.001) with no significant interaction effect of group (*F*_4.706, 97.328_ = 0.821, *p* = 0.537). A comparison of between subject effects found significant group effects (*F*_2, 15_ = 4.544, *p* = 0.03; **p < 0.01, Fisher's LSD) but there was no difference between blast-treated with minocycline and blast-treated with vehicle (*p* = 0.213). Error bars indicate the SEM in all panels.

When rats were tested in a cued fear learning protocol, all groups trained similarly exhibiting freezing in response to pairing the tone and the shock (Figure 4A). However, in the cued testing (Figure 4B), the blast-exposed groups, whether treated with vehicle or minocycline, froze more the sham + vehicle, and there was no difference between blast-treated with minocycline and blast-treated with vehicle (*p* = 0.213). Thus, while a course of minocycline given five times over 9-day period improved blast-induced deficits in object recognition memory and anxiety, exaggerated fear leaning was not improved.

### A 4-week course of minocycline reverses blast induced anxiety and cognitive deficits but not exaggerated fear learning

**Figure 5:**
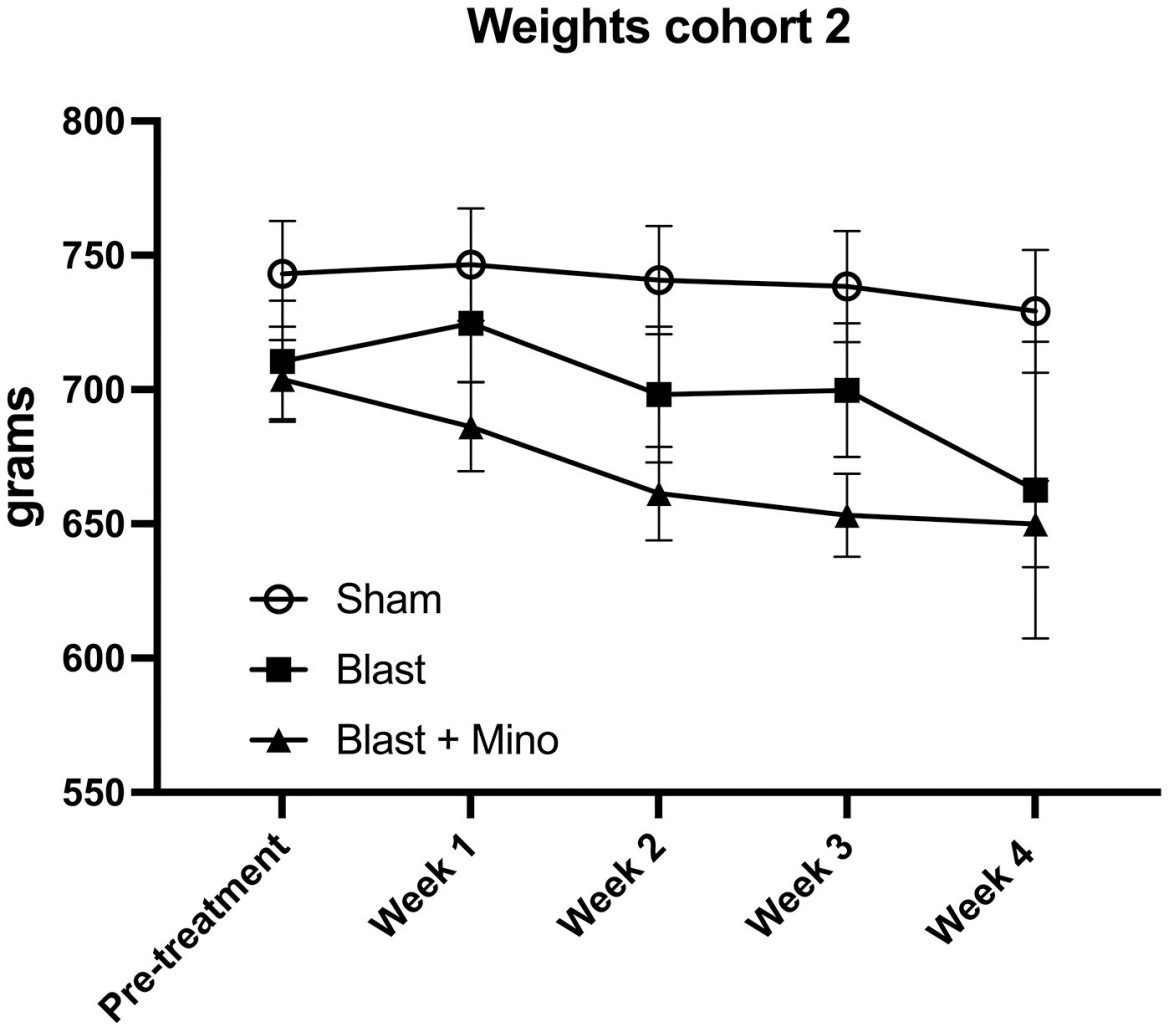
Weights for cohort 2. Shown are the weights for the rats used in cohort 2, obtained before and at the end of treatment. A repeated measures ANOVA showed that rats lost weight comparing before to after treatment (*F*_1.303, 48.226_ = 5.265, *p* = 0.018) but there was no interaction effect of group (*F*_2.607, 48.226_ = 1.182, *p* = 0.323) and no between group differences (*F*_2, 37_ = 2.723, *p* = 0.079). Error bars indicate the SEM.

Reasoning that treatment for 5 days over a 9-day period might be sufficient to rescue some traits but not others, we designed a treatment protocol in which rats received three treatments per week with the first administration on a Wednesday and then continued Monday, Wednesday, and Friday over a period of 4 weeks (11 administrations total, Figure 1). The weights for cohort 2 before treatment and during the protocol are shown in Figure 5. There were no between group differences (*F*_2, 25_ = 2.537, *p* = 0.099) and there was no mortality during the study. At the end of treatment rats were tested in an EZM, NOR and cued fear learning. Rats received one additional booster dose of drug or vehicle between NOR and cued fear testing (Figure 1).

**Figure 6:**
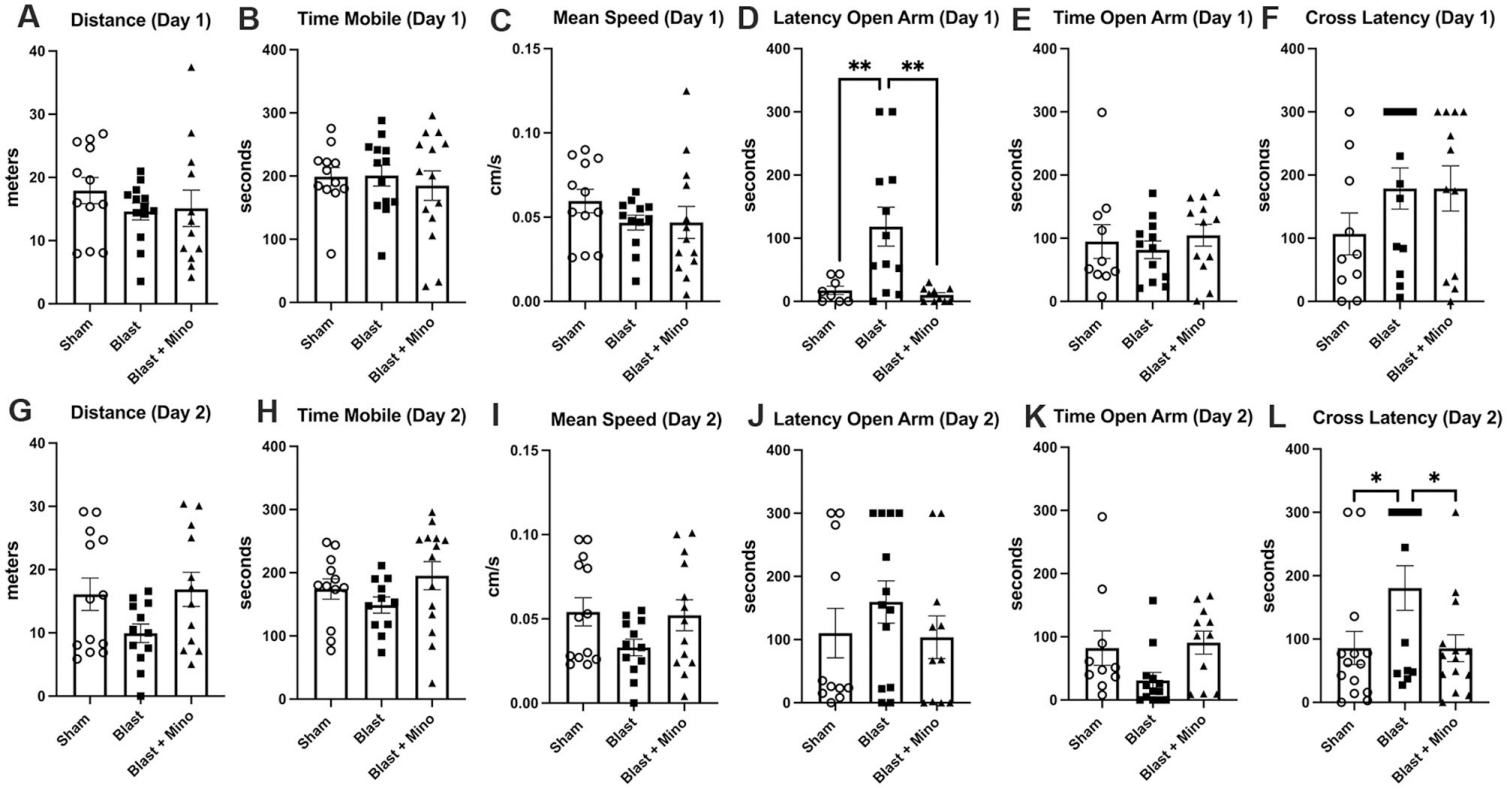
Testing of cohort 2 in EZM. Blast-exposed and control rats were tested for 5 min on two consecutive days in an EMZ. Shown is total distance moved **(A, G)**, time in motion **(B, H)**, mean speed **(C, I)**. latency to enter an open arm **(D, J)**, time spent in an open arm **(E, K)** and the latency to cross between two open arms (**F**, **L**, cross latency) on days 1 and 2. Error bars indicate the SEM in all panels. One-way ANOVAs were significant for open arm latency on day 1 (*F*_2, 27_ = 8.295, *p* = 0.0016) and cross arm latency on day 2 (*F*_2, 38_ = 3.795, *p* = 0.0.0314). Asterisks indicate values significantly different between groups (*p < 0.05, **p < 0.01, Fisher's LSD).

EZM was tested on two consecutive days (Figure 6). Unfortunately, the anxiety phenotype was not as strong in the blast-exposed treated with vehicle as has been observed in many prior studies (5, 6, 8, 9, 38, 41). However, the two measurements that were abnormal when comparing blast-treated with vehicle to sham treated with vehicle (latency to an open arm day 1, cross latency day 2) were both reversed by minocycline.

**Figure 7:**
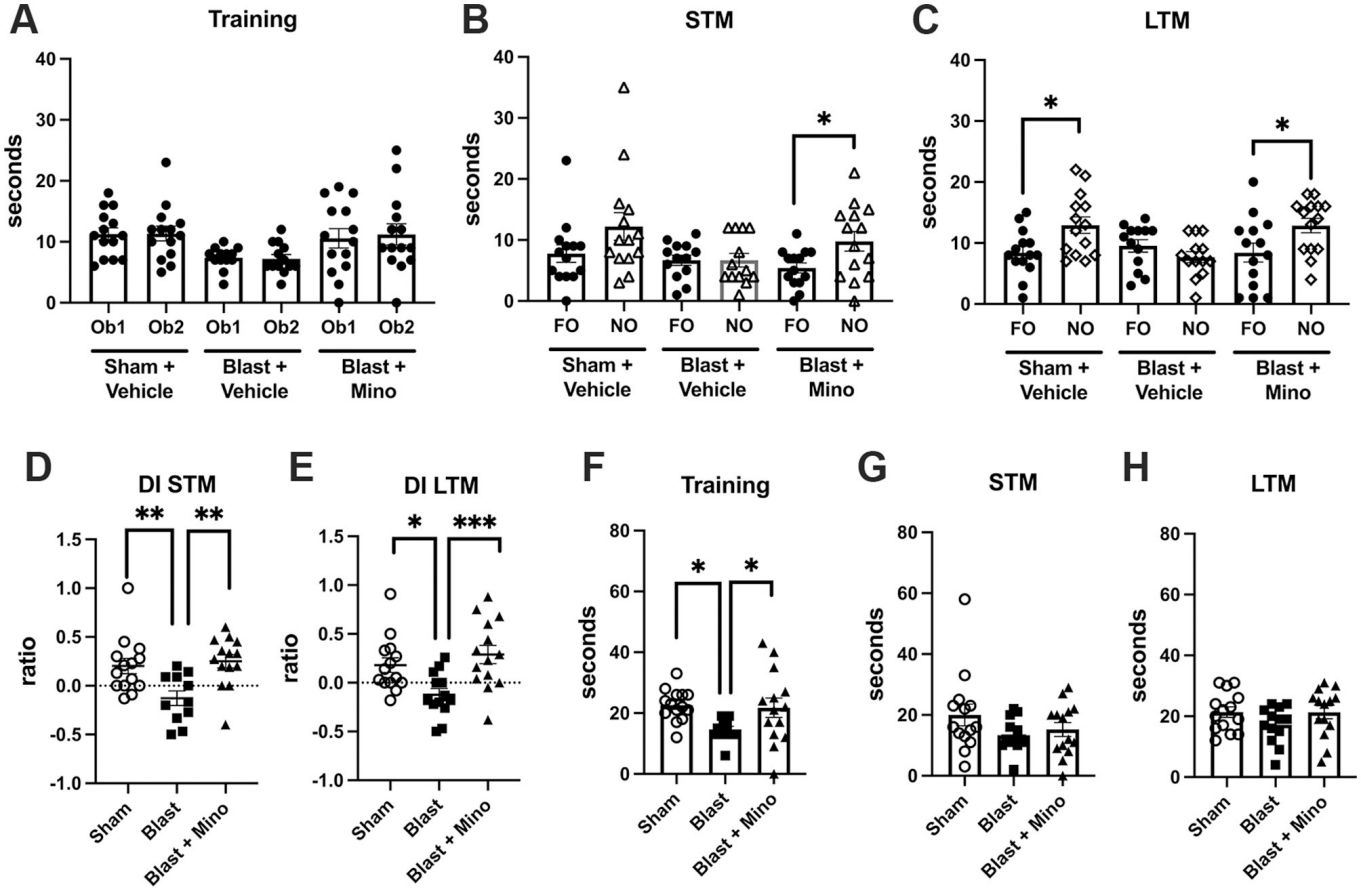
Testing of cohort 2 in NOR. Shown is time spent exploring object 1 (Ob1) or object 2 (Ob2) during the training **(A)**, short-term memory testing (STM) at 1 h after training **(B)** or long-term memory testing (LTM) at 24 h after training **(C)**. Time spent exploring the familiar (FO) and novel (NO) objects were compared using unpaired *t*-tests (*p < 0.05). Panels **D** and **E** show discrimination indices (DI) calculated for the STM **(D)** and LTM **(E)** testing. Panels **(F–H)** show total exploration time during the training **(F)**, STM **(G)** and LTM **(H)** testing. One-way ANOVAs were significant for STM DI (*F*_2, 36_ = 7.117, *p* = 0.0025), LTM DI (*F*_2, 38_ = 7.112, *p* = 0.0024) and exploration during training (*F*_2, 38_ = 4.168, *p* = 0.0231). Asterisks in panels **(D–H)** indicate significant differences between groups (*p < 0.05, **p < 0.01, ***p < 0.001 Fisher's LSD). Error bars indicate the SEM in all panels.

In NOR testing of cohort 2 during training all groups spent equal amounts of time exploring the objects (Figure 7A). In STM testing blast + minocycline explored the NO more than the FO while sham and blast + vehicle explored them equally (Figure 7B). In LTM testing, sham + vehicle and blast + minocycline explored the NO more than the FO while blast + vehicle explored them equally (Figure 7C). When a DI was calculated for the STM (Figure 7D) and LTM (Figure 7E) testing, deficits in object recognition in the blast + vehicle were rescued by minocycline in the blast-exposed in both. Blast +vehicle also spent less total time exploring the objects, an effect that was restored in blast + minocycline (Figure 7F). All groups spent equal total time exploring the objects in the STM (Figure 7G) and LTM (Figure 7H) testing.

**Figure 8:**
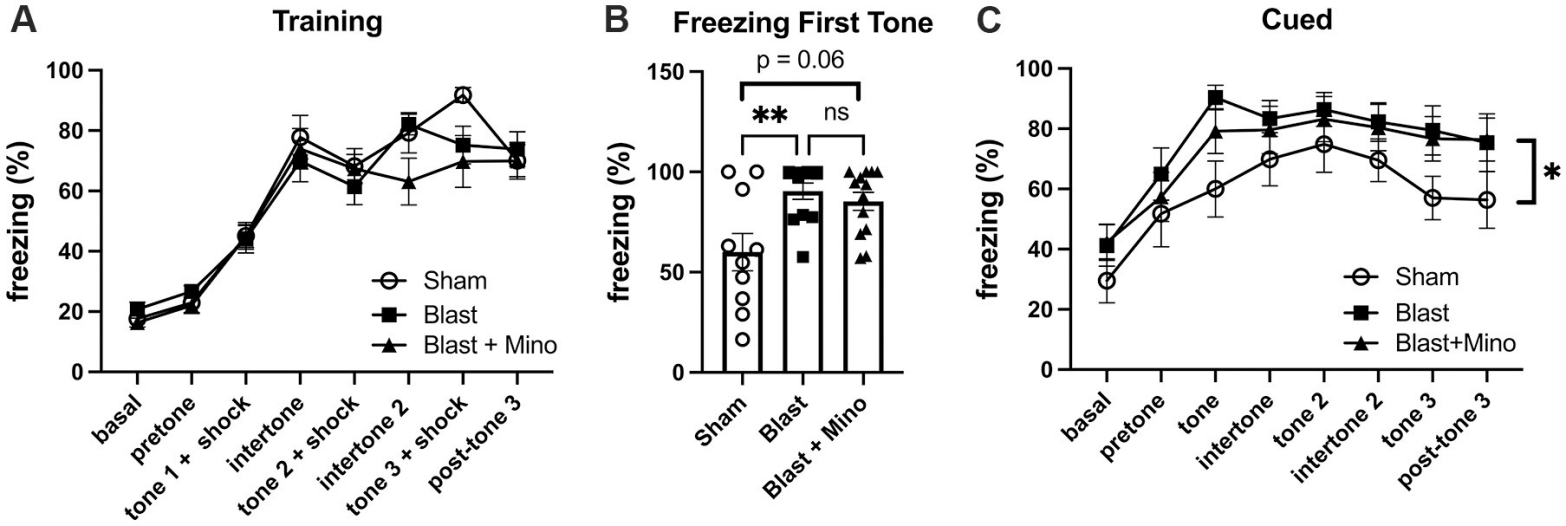
Testing of cohort 2 in fear learning. Panel **(A)** shows training. A repeated measures ANOVA showed that all groups responded with increasing freezing following pairing of the tone and shock (*F*_4.300, 159.098_ = 159.098, *p* < 0.001) with no interaction effect of group (*F*_8.600, 159.098_ = 1.705, *p* = 0.095) and no between group differences (*F*_2, 37_ = 0.721, *p* = 0.493). Panel **(B)** shows a comparison of freezing to the first tone during the cued phase testing. A one-way ANOVA was significant (*F*_2, 32_ = 6.8, *p* = 0.0034). *p* Values are indicated (**p < 0.01; ns, not significant). During the cued testing session **(C)**, all groups responded with freezing following presentation of the tone (repeated measures ANOVA; *F*_2.949, 97.328_ = 18.994, *p* < 0.001) with no significant interaction effect of group (*F*_5.899, 97.328_ = 0.455, *p* = 0.837). A comparison of between subject effects found that blast froze more than sham (*p* = 0.05) but there was no difference between blast-treated with minocycline and blast-treated with vehicle (*p* = 0.646). Error bars indicate the SEM in all panels.

When cohort 2 was tested in fear learning (Figure 8), as in the first cohort, all groups responded during training (Figure 8A) with increasing freezing following pairing of the tone and shock and there were no differences between groups. However, also as in cohort 1 in cued fear testing the blast-exposed groups whether treated with minocycline or not, froze more than the sham when the tone was presented without the shock and this effect was not rescued by minocycline (Figures 8B, C). Thus, while a 4-week course of minocycline rescued blast-induced deficits in object recognition and anxiety, as in the first experiment it did not rescue exaggerated fear learning.

### Minocycline does not alter blast-induced effects on serotonin 2A receptor (5-HT2AR) or post-synaptic density protein 95 (PSD-95) expression

**Figure 9:**
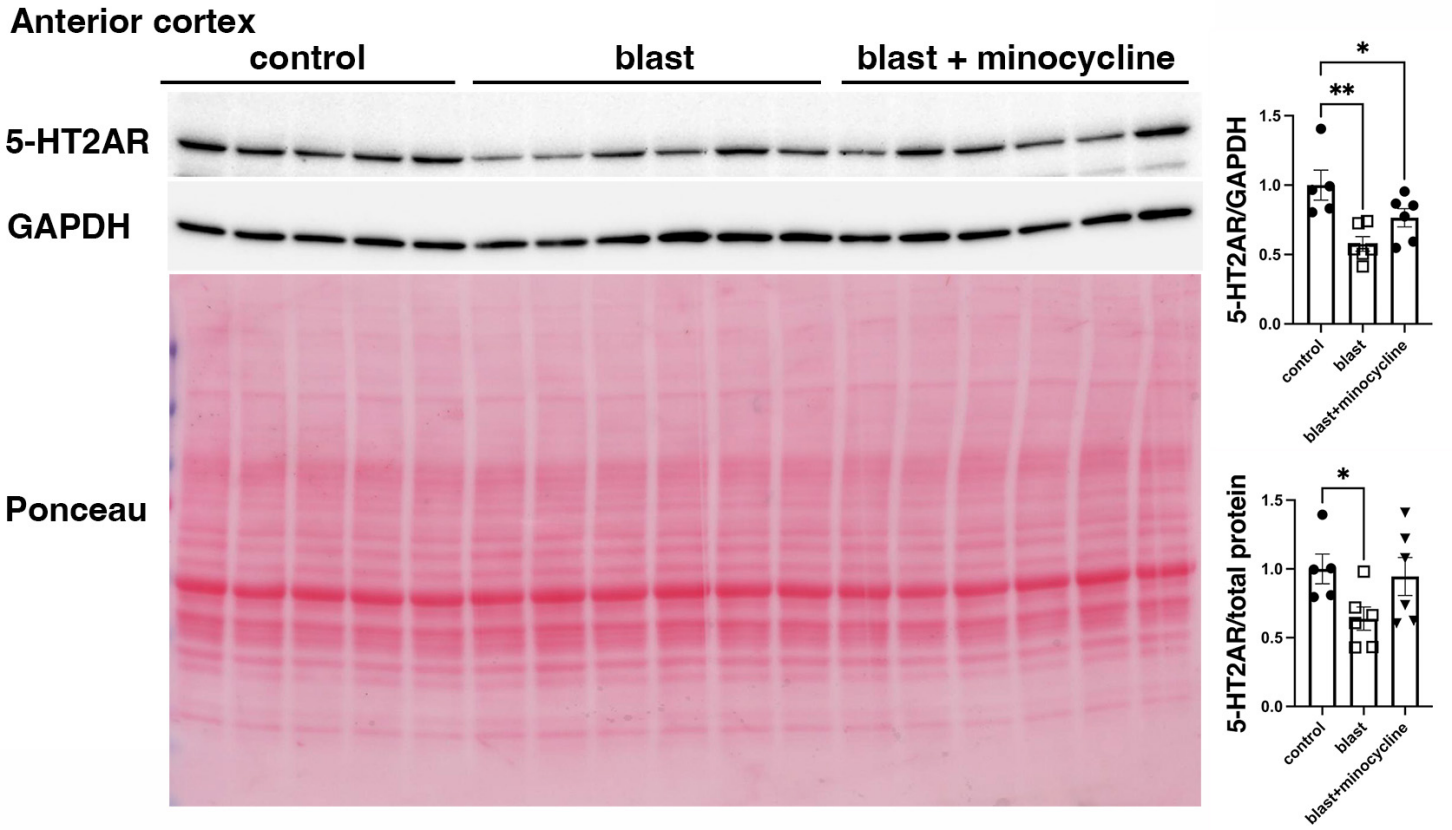
Western blot analysis of 5-HT2AR in rats from cohort 2. 5-HT2AR expression was analyzed in the anterior cortex of control rats treated with saline (*n* = 5), and blast-exposed rats treated with saline or minocycline (*n* = 6). Top panel: 5-HT2AR; middle panel GAPDH; bottom panel: Ponceau S staining. The targets were sequentially analyzed on the same blot in the same order as indicated in the Figure. The bar graphs show 5-HT2AR levels expressed as a ratio to GAPDH or total protein load calculated from the Ponceau S staining. Error bars indicate the SEM in all panels. Asterisks indicate significant differences (Fisher's LSD after a significant one-way ANOVA, *p < 0.05, **p < 0.01).

**Figure 10:**
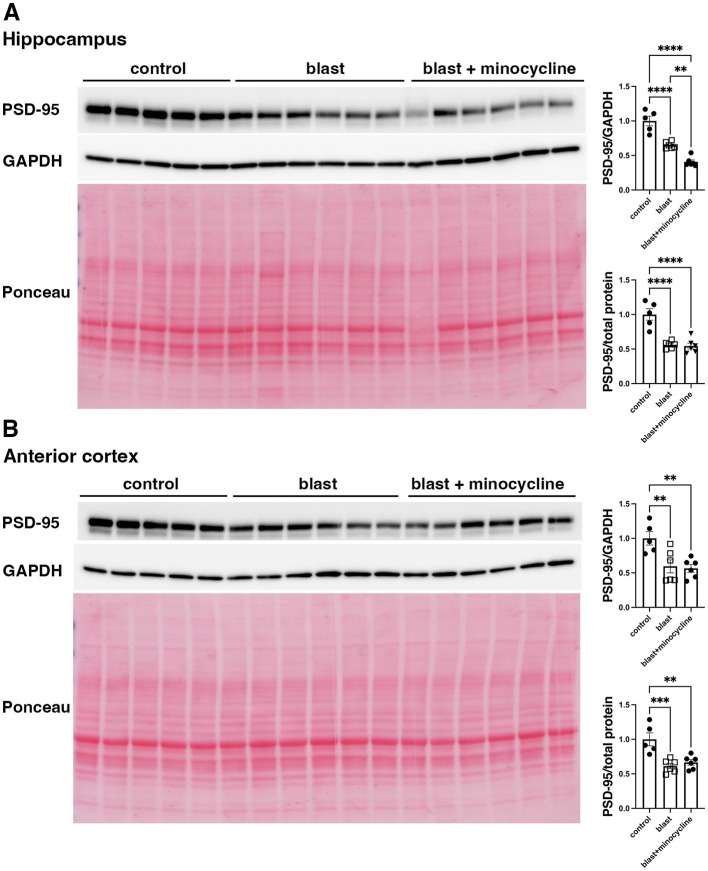
Western blot analysis of PSD-95 in rats from cohort 2. PSD-95 was analyzed in hippocampus **(A)** and anterior cortex **(B)** of control rats treated with saline (*n* = 5), and blast exposed rats treated with saline (blast) or minocycline (*n* = 6). Top panel: PSD-95; middle panel: GAPDH and bottom panel Ponceau S stain. The targets were sequentially analyzed on the same blot in the same order as indicated in the Figure. The bar graphs show PSD-95 levels expressed as a ratio to GAPDH or total protein load calculated from Ponceau S staining. Error bars indicate the SEM. Asterisks indicate significant differences (Fisher's LSD after a significant one-way ANOVA, **p < 0.01, ***p < 0.001, ****p < 0.0001).

To begin to explore the underlying mechanisms of minocycline's effects on at least some blast induced behavioral traits we examined expression of 5-HT2AR and PSD-95 which have both been found to be decreased after blast exposure in this model (42, 43). In studies on animals from cohort 2, while 5-HT2AR (Figure 9) and PSD-95 (Figure 10), were decreased following blast exposure minocycline did not reverse these changes.

### Minocycline modulates microglial morphology

**Figure 11:**
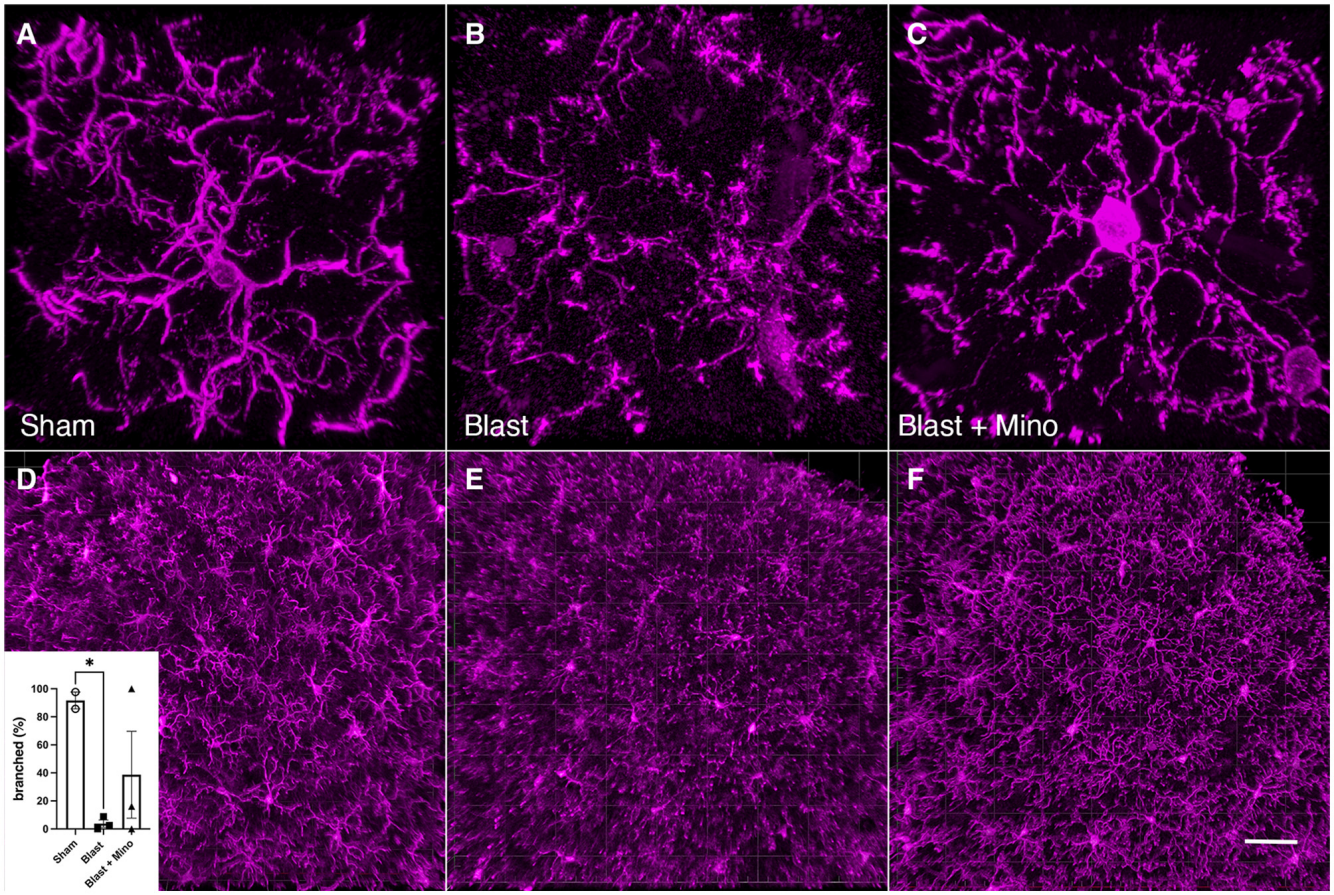
Minocycline effects on microglial morphology. Shown are high and low magnification 3D images of microglia (Iba1^+^, magenta) in the somatosensory cortex of control treated with saline (**A** and **D**), blast-exposed treated with saline (**B** and **E**) and blast-exposed treated with minocycline (**C** and **F**). Inset in panel **(D)** shows the percentage of Iba1+ microglia which were branched (as in panels **A** and **C**) vs. unbranched (panel **B**). Cells were scored by an observer blinded to the condition of the animal (*n* = 2 sham, *n* = 3 blast; *n* = 3 blast + minocycline). A mean of 48.6 ± 4.2 (SEM) Iba1 positive cells were scored per animal. Error bars indicate the SEM (*p < 0.05). Animals are from cohort 2. Scale bar: **A–C**, 10 μm; **D–F**, 50 μm.

To determine whether minocycline might be modulating microglial activation state, we stained tissue sections from cohort 2 with Iba1. As shown in Figure 11, compared to the branched morphology of microglia in sham animals (Figures 11A, D), blast exposed without treatment frequently exhibited an activated ameboid transition with an enlarged soma and decreased branching (Figures 11B, E). By contrast microglia in blast-exposed rats treated with minocycline (Figures 11C, F) exhibited a more branched morphology (quantitated in panel D).

## Discussion

We used a well-established animal model which employs male rats and mimics the type of open field low-level blast exposure associated with human mild traumatic brain injury (mTBI) or subclinical blast exposure in humans (5–9, 19–22, 34–38). The pressure level used (74.5 kPa, 10.8 psi) is transmitted to brain (44) but does not cause gross neuropathological effects nor lung injury (40). Because blast-related TBI may involve a combination of injuries related to effects of the primary blast wave as well as damage from rotational/acceleration injury (45, 46), during the overpressure exposures head motion is restricted to minimize rotation/acceleration injury. A three-exposure paradigm was chosen to mimic the multiple blast exposures that are commonly experienced by Service Members in modern military environments (47, 48).

Inflammation has long been suspected of playing a role in blast-induced brain injury (16, 17). The purpose of this study was to determine whether minocycline, an anti-inflammatory and neuroprotective agent, could reverse blast induced behavioral traits after they are established by administering minocycline to male rats at 8–8.5 months after blast exposure when the behavioral phenotype is established.

In an initial study minocycline was given five times over a 9-day period. This regimen improved blast induced deficits in object recognition memory and anxiety but did not rescue exaggerated fear leaning. Reasoning that treatment for 5 days over a 9-day period might be insufficient to rescue all traits, we conducted a second study in which rats received a total of 11 administrations of minocycline over a period of 4 weeks. The results of this study were similar to the original; object recognition memory and anxiety were rescued but exaggerated fear leaning was not. Thus, in two studies aspects of the behavioral phenotype were readily reversed while others were not.

The rapid reversal of the cognitive and anxiety related phenotype in comparison to the resistance of the fear leaning phenotype suggests that different mechanisms may be driving the two phenotypes. Rapid reversal of cognitive and anxiety related traits suggests that these are being driven by levels or combinations of cytokines/inflammatory factors which can be modulated by minocycline. By contrast, traits such as exaggerated fear learning may be independent of inflammation or if the result of inflammation, associated with chronic structural changes that are not readily reversible once established.

In an initial series of studies designed to probe the effects of minocycline on blast related injury we did not find any rescue of 5-HT2AR or PSD-95 expression which have been found to be decreased after blast exposure in this model (42, 43). Minocycline has been frequently reported to inhibit microglial activation and we found very similar changes reported in other studies (49–51) in terms of minocycline's effects on microglial morphology. Blast exposed rats without treatment frequently exhibited a proinflammatory ameboid morphology which was partially restored to the branched morphology of controls by 4 weeks of minocycline treatment. Due to the small sample size and variability in the minocycline treated animals, these results must be interpreted with some caution and future studies will need to be performed on larger sample sizes. However, they provide some support for the notion that whatever minocycline's beneficial effects are they are at least in part being mediated through actions on microglia. How this observation would explain minocycline's effect on cognition but not fear learning is unclear. Future studies will be needed to understand minocycline's effects on inflammatory cytokines and other factors modulated by microglial inflammatory state.

TNFα has been elevated in many studies following blast injury in rodents (16, 52–54), Soldiers exposed to moderate blasts during training exercises show transient increases in blood TNFα levels (55). Human studies in experienced breachers exposed to high numbers of career blast exposures also show dysregulation of genes associated with chronic inflammation in blood (56) and have identified elevated TNFα levels in neuronal-derived extracellular vesicles isolated from serum (57). TNFα is a therapeutic target in many human inflammatory conditions and has revolutionized treatment of some (58).

A prior transcriptomic study in our rat model identified TNFα as a potential upstream regulator of a cluster of differentially expressed genes whose expression was affected by blast exposure (22). TNFα is of special interest in that minocycline has broad anti-inflammatory and neuroprotective actions (23) with multiple studies documenting its ability to inhibit TNFα production (24–30). In brain, TNFα has both homeostatic and pathological roles. It is produced mainly by microglia but also by neurons and astrocytes (59). Under physiological conditions, constitutive TNFα expression plays roles in synaptic plasticity, learning and memory (59). Under pathological conditions, microglia and astrocytes release TNFα which is a crucial mediator of chronic inflammation and secondary brain injury (60). TNFα in brain also has biological activities independent of its role in inflammation, being implicated in PTSD-related hyperarousal states and regulating fear learning (17).

Thus, a variety of evidence supports a potential role for TNFα signaling in the neurobiological basis of blast effects on behavior through inflammatory and non-inflammatory mechanisms although in this study minocycline did not reverse blast effects on fear learning. Exaggerated fear responses in this model have been reversed by mGluR2/3 antagonists and hydroxynorketamine (9, 22, 38), agents that would be expected to act on already established neurochemical abnormalities and likely to act independent of inflammation. Future studies will be needed to determine the extent to which minocycline's effects on TNFα activity may underlie its partial effectiveness on the blast-related behavioral phenotype.

However, it should be noted that while minocycline has TNFα inhibiting activity, it also has broader anti-inflammatory and non-inflammatory effects, including affecting calcium regulation, mitochondrial stabilization and reducing oxygen radical toxicity (61). This model develops a chronic tau pathology (34) and minocycline reduced tau pathology in a rat model of neuroinflammation (62). Chronic alterations in perivascular microglial and astrocytes are seen in the current model (20, 21, 43, 63). These cells might also be targets for minocycline's actions independent of any TNFα effects.

Several limitations of the current study should be mentioned. One is the lack of inclusion of female rats. Sex differences in TBI outcomes are well-known (64) with studies in female Veterans suggesting that they are more likely to report persisting neurobehavioral symptoms and use more outpatient services than their male counterparts (65). In experimental animals, sex differences in response to blast has been little studied, although several reports have suggested that blast responses in female rats and mice differ (66–68). With the increasing number of female Veterans these studies assume a high importance. Another limitation is the lack of insight these studies provide at the molecular level into how minocycline is reversing some behavioral deficits but not others. Future studies will be needed to address other mechanisms.

## Conclusion

Our findings demonstrate that minocycline treatment can rapidly reverse cognitive changes and anxiety but not exaggerated fear learning. These studies have implications for understanding the nature of blast-induced behavioral traits, some of which may result directly from an inflammatory environment that can be rapidly reversed while others may be independent of inflammation, or not reversible once structural or neurochemical changes are established. Future prevention studies seem warranted to determine whether initiating anti-inflammatory therapy before symptom onset can block the development of the full behavioral phenotype. In summary, while inflammation appears to play a significant role in the pathogenesis of blast-induced behavioral changes, it is likely only one component of a complex and multifaceted process.

## Data Availability

The raw data supporting the conclusions of this article will be made available by the authors, without undue reservation.

## Author's note

The views expressed in this article reflect the results of research conducted by the authors and do not necessarily reflect the official policy or position of the of The Henry M. Jackson Foundation for the Advancement of Military Medicine, Inc., the Department of the Navy, or the Department of Defense (DoD). Mention of trade names, commercial products, or organizations does not imply endorsement by the U.S. Government. The study protocol was reviewed and approved by the Institutional Animal Care and Use committees of the Walter Reed Army Institute of Research/Naval Medical Research Command and the James J. Peters VA Medical Center. Studies were conducted in compliance with all applicable Federal regulations governing the protection of animals in research and in compliance with the Animal Welfare Act and per the principles set forth in the “Guide for Care and Use of Laboratory Animals,” Institute of Laboratory Animals Resources, National Research Council, National Academy Press, 2011. Some of the authors are military Service Members or federal/contracted employees of the United States Government. This work was prepared as part of their official duties. Title 17 U.S.C. § 105 provides that Copyright protection under this title is not available for any work of the United States Government. Title 17 U.S.C. § 101 defines a U.S. Government work as a work prepared by a military Service Member or employee of the U.S. Government as part of that person's official duties.
